# Impact of exploration behavior, aptitude for pellet consumption, and the predation practice on the performance in consecutive live prey foraging tests in a piscivorous species

**DOI:** 10.1007/s10071-023-01747-4

**Published:** 2023-01-28

**Authors:** Tamás Molnár, Béla Urbányi, Ildikó Benedek

**Affiliations:** 1 Department of Molecular Ecology, Institute of Aquaculture and Environmental Safety, Hungarian University of Agriculture and Life Sciences, Guba S. Street 40, Kaposvár, 7400 Hungary; 2 Department of Aquaculture, Institute of Aquaculture and Environmental Safety, Hungarian University of Agriculture and Life Sciences, Gödöllő, Hungary; 3 Department of Animal Breeding, Institute of Animal Breeding Sciences, Hungarian University of Agriculture and Life Sciences, Kaposvár, Hungary

**Keywords:** *Sander lucioperca*, Pikeperch, Neophobia, Prey detection

## Abstract

Within the predator–prey relationship, predator behavior is less studied. Even in natural populations, it shows great diversity, and the factors influencing this are even less known. Among these factors, the personality type of the individual, (including exploration, and neophilia) and the practice significantly influence the success of adapting to a changing environment and switching to new prey types. In the present study, we investigated the first five consecutive foraging trials on live fish prey in naïve pikeperch individuals, which previously consumed or refused pelleted food. We hypothesized that individuals which were willing to consume alternative (pelleted) food would also show higher foraging success on living prey and that the practice would influence the learning process. Our results show that the timing of prey detection is influenced by exploratory behavior, the latency of the first attack by the aptitude for consuming pellets, and both traits by the individual's practice. However, neither of the factor affects the latency and success rate of capturing the prey, suggesting that predation is an independent behavioral trait.

**Supplementary Information:**

The online version contains supplementary material available at 10.1007/s10071-023-01747-4.

## Introduction

The environment shows changes by anthropogenic influences which have a strong effect on the behavior of individuals, causing an adaptation in which cognition has a significant role (Griffin et al. 2017). In fish, an example of this is the change in natural prey acquisition following the controlled rearing of the offspring of commercially exploited species (Brown and Day 2002). The inefficient foraging behavior of these individuals in nature, as it is partly an innate trait, is probably due to inexperience with natural food, which can be counterbalanced within the framework of food training (simple exposure to natural food) before stocking (Näslund 2021). Foraging ability, like other behavior, can be enhanced by learning from individual experiences and peers (Strand et al. 2010). The latency of the first attack in brown trout was longer in hatchery individuals when compared to wild ones, but this respective difference decreased with practice. Additionally, foraging efficacy was higher in wild individuals and increased with practice (Sundström and Johnsson 2001). The level of post-release mortality depends on the capability of the fish to switch foraging from predictable pellet feeding to live, moving prey. The ability to learn determines the speed of improvement of the handling skills for different prey types (Braithwaite 2006). The lower prey capture success in pellet-raised individuals is probably the result of inadequate prey capture kinematics (technique), using slower kinematics with smaller excursions (Wintzer and Motta 2005), making fewer attempts to feed, and having longer delays in the time (latency) to strike (Caldentey et al. 2021). However, the technique is quickly improved by learning during consecutive live prey exposure trials (Wintzer and Motta 2005; Caldentey et al. 2021). The fish’s performance shows a learning curve; it is initially slow, then rapidly improves to a point where it no longer exhibits faster predation behavior (Braithwaite 2006). Experience not only results in faster catching of prey but also leads to energetic gains by changing attack latency and capture efficiency (Dill 1983).

The studies on foraging behavior mainly focus on the features of the prey in the predator–prey relationship; how prey can avoid predation, and how the characteristics of prey individuals (physiological, behavioral, etc.) affect it (Belgrad and Griffen 2016; Szopa Comley et al. 2020). From the predator aspect, the relationship is less explored, and the behavior of the predator is often considered constant in the studies (Lima 2002). In the last decade, behavioral syndromes (“a suite of correlated behaviors expressed either within a given behavioral context or across different contexts” Sih et al. 2004) were described (similarly to prey) also in the predator species and the behavior types were found to vary widely within the natural populations.

The low-stimulus environment (lack of predators, predictable food supply) often results in the advantage and fast growth rates of aggressive and explorative individuals, under hatchery conditions (e.g., in rainbow trout *Oncorhynchus mykiss* and brown trout *Salmo trutta)* (Ward et al. 2018). Although rapid growth allows the individual to have better access to food sources, an energy allocation trade-off between the brain and the digestive tract may decrease in the cognitive performance of captive fish (Salena et al. 2021).

The pace-of-life syndrome (POLS) hypothesis originally explained population or species differences in suites of life history and physiological traits, extending the concept of a fast–slow life history continuum (Dammhahn et al. 2018). Reale et al. (2010) adaptively integrated the behavior into this hypothesis, connecting personality traits with physiological properties (e.g., immune system, stress sensitivity). The two extremes for personality traits related to the slow–fast continuum characterize proactive (fast, bold, highly active) and reactive (slow, shy, less active) individuals with different metabolism. Sih and Del Giudice (2012) extended POLS with the inclusion of cognition, based on the linkage between the fast–slow behavioral types and the cognitive speed–accuracy trade-offs. The notion of “cognitive syndrome” suggests that individuals also show diversity based on problem-solving (learning) ability, and this trait is related to their behavior type. Flexible individuals who respond rapidly to environmental change are generally cautious and reactive, while bold, proactive individuals are more routinized and predictable in their behavior over time (Jolles et al 2019). Although fast individuals make decisions quickly, they make more mistakes, make less use of their experience due to being insensitive to the new information, and are slow at reversal learning, especially in tasks requiring reduced activity (Sih and Del Giudice 2012).

The target species of the present investigation, the pikeperch is a large percid fish with high ecological and economical importance. From the three Eurasian Sander species, it has the largest habitat range in central Europe and western Asia and has been introduced to European countries (Kottelat and Freyhof 2007). Similarly to other percid fish, it follows ontogenetic patterns in diet, shifting from zooplankton to piscivory. However, pikeperch is an obligate piscivore, shifting to piscivore earlier compared to other species, and cannibalism often occurs as a population regulatory factor (Feiner and Höök, 2015). Under laboratory conditions, the shift starts with the size of 11.0 mm, and, at the size of 19.9 mm, 90% of the stock is already piscivorous if the prey is available (Colchen et al. 2020). In nature, obligate piscivority can be observed in 13.5 mm fish (Specziár, 2011), but fully develops in fish of 35–100 mm or longer (Ginter et al. 2011; Mittelbach and Persson 1998; Specziár, 2011). This process is coupled with morphological and physiological changes that happen quickly (within three weeks) and shows great individual variability in pikeperch.

During the tank rearing of the species, switching from zooplankton to food with a higher energy content is utilized when habituating it to pelleted food. This adaptation had a selective effect on the behavior of pikeperch (Molnár et al. 2018). The speed of the learning pellet consumption resulted in different behavior types: the early-switching individuals were less explorative and stress-sensitive, in contrast to the late- and non-switching groups, which consisted predominantly of explorative individuals with lower cortisol responses. The ecological impact of intensive rearing has not been studied in the species. Even though intensively (in tanks) produced pikeperch fingerlings can be adapted to pond culture when prey fish species are available, their survival in ponds shows varying levels. It is similar to the value of stocks originally reared in ponds if the pikeperch is the only predator species in the ponds (65.2% (Blecha et al. 2016) and 84.3% (Zakęś et al. 2015)), but it decreases significantly in the presence of other predators (22.9% (Zakęś et al. 2015)). However, whether this increased mortality in the presence of other predator species is a result of their decreased competitive ability (poorer predatory performance) or not adequate predator avoidance (higher predation rate on the more exploratory individuals) has not yet been demonstrated.

In the present study, we hypothesized that based on the “cognitive syndrome”, pellet-consuming individuals are less explorative, and cautious but more flexible and show higher foraging success on living prey. In our study, we investigated the latency of elements of predatory behavior (detection, first attack, successful predation) and the method of prey capture. Our primary question was whether individuals that are faster at learning to eat pellets also perform faster on live prey and, if so, which elements of prey acquisition are affected. Our secondary objective was to see whether, as in the previous study, pellet-consuming individuals would be less exploratory and whether exploration would affect live prey capture independently of pellet consumption. Our third objective was to determine the number of trials required to reach a threshold during learning, after which prey capture latency is no longer significantly reduced.

## Materials and methods

### Ethical approval

All procedures performed in this study followed the European Directive 2010/63 UE. The protocol was in accordance with the ethical standards of the Hungarian University of Agriculture and Life Sciences Kaposvár Campus and approved by the Committee on the Ethics of Animal Experiments of the Hungarian University of Agriculture and Life Sciences Kaposvár Campus (permit number: 3/2016-MÁB). The authors confirm that all experiments were performed in accordance with relevant guidelines and regulations.

### Culture facility

The rearing unit, previously used by Molnár et al. 2018, contained 30 aerated aquaria with a volume of 65 L (60 × 30 × 30 cm, L × W × H). These were parts of a recirculation system. This system had a total volume of 2600 L (attached to a simple bio-filter unit). The walls of the aquaria were painted black except for the front side. The daily water replacement rate was about 10% of the total volume. The water flow rate was adjusted to 1.5 L min^−1^, and the temperature was kept at 21 ± 0.5 °C. The lighting provided 50 lx with a cycle of 12/12 h. The water quality parameters were determined twice a week using a Compact Photometer PF-12 Plus (Macherey–Nagel). The measured parameters were as followings: dissolved oxygen = 7.8 ± 0.4 mg L^−1^, pH = 8.0 ± 0.1, ammonia (NH_4_^+^) = 0.3 ± 0.2 mg L^−1^, and nitrite (NO_2_^−^) = 0.15 ± 0.03 mg L^−1^.

### Experimental fish

Juvenile pikeperch (*Sander lucioperca* L.) (mean standard length 38.4 ± 0.6 mm, mean body weight 0.85 ± 0.16 g, *N* = 2000) were purchased from the BO-FA Fish Farm (Attala, Hungary). The habituation process was very similar to the protocol published by several authors (Bódis et al. 2007; Policar et al. 2013, 2016). In this procedure, 7 to 12 days after habituation starts, the majority of the fish consume pellets. On the 12th day, approximately 80% of the fish survive, and the rest died due to starvation. To avoid this mortality, a small amount of *Tubifex* was offered as maintenance feed once every day after the mix of *Tubifex* and pellet was offered. Pellet feeder individuals were determined on the 21st day of the trial: after the removal of cannibal individuals, only pellets were offered until the fish accepting the pelleted food were satiated. Pellet consumption was recognized by the larger size and yellow configuration of the abdomen. The individuals refusing the pellet were separated and *Tubifex* was offered to them.

During habituation, fish were kept in eight 300 L aerated aquaria (the fish density was 0.8 individuals/L). The aquaria worked in a recirculation system with a total volume of 3000 L and were equipped with a UV filter. The size of the pellets was 1.1 mm (Nutra Pro 2.0, 54% protein, 18% fat) during habituation and changed during the following rearing period (10 weeks) between 1.5 and 4 mm: Nutra Pro MP-L, 1.5 mm (54% protein, 20% fat), Alterna 1P, 2.5 mm (47% protein, 20% fat) and Alterna 2P, 4 mm (45% protein, 20% fat). The fish consumed the pelleted food, Skretting Alterna 2P, before the start of the live prey foraging tests, while fish refusing to consume the pellet continued to consume *Tubifex*.

Continuous detection and removal of cannibalistic individuals were performed during the experiment. Cannibals were easily detectable in the morning before feeding on their full stomach (often the tail of the peer hanging out of the mouth of the cannibal), and these individuals were removed and reared in a separate tank. Cannibals were excluded from the behavior test.

From the total of 36 fingerlings with an average standard body length of 90.0 ± 3.2 mm, two sub-groups were formed: 18 that consumed pellets (Pellet Feeder-PF; 90.0 ± 2.8 mm) and 18 that did not (pellet non-feeder (PNF); 90.0 ± 3.6 mm).

The survivors of the experiment were used in further experiments after the study, as the natural behavior of the animals was studied. We did not use euthanasia.

### Behavior tests

#### Novel object test in a novel environment

A novel object test was performed according to Molnár et al. (2018). The fish were transferred into test tanks (50 cm × 30 cm × 30 cm, L × W × H) individually. Following 24 h of habituation, a video camcorder (Sony HDR-XR) was started and a small yellow Lego block (LEGO 6176 DUPLO Basic Brick) attached to a metal sinker was placed in the center of the tank as a novel object and the behavior of the fish was recorded for 30 min. The number of approaches (frequency) to the novel object, the closest distance from the object (Dmin), and latency of the closest approach (Tmin) were estimated.

#### Live prey foraging test

12 h preceding the tests, the fish were transferred individually into the test aquarium. For every single individual, five consecutive hunting tests were conducted, and 12 h breaks were applied between the tests to maintain motivation (hunger). Animals from the two groups were tested in parallel in a random order, the test aquariums were randomly used for the groups.

Three sides of the test aquarium (90 × 35x35 cm) were painted black. The aquarium contained small-sized, dark, granulated gravel and an artificial plant. It was divided by a non-translucent plate. The tested pikeperch was placed into the larger part (70 cm), while the smaller part contained three premature (not colored) Rosy Barb (*Pethia conchonius*) fish as prey with a standard length of 20 mm. The Rosy Barbs were bred for this purpose, and in each session, new (naive) individuals were used. The test started with the removal of the separating plate and ended with a successful hunting attempt or terminated after 60 min. The behavior of the tested pikeperch was recorded with a Sony HDR-XR camera. During the experiment, a total of 180 records from 36 fish were analyzed. The video recordings were analyzed manually with the video trimming function of the PMB version 5.2 software. The same person detected the behavioral events for all fish, and the video ID did not contain any information about the treatment groups. While evaluating the video, the following variables were recorded: the time points (TP) of 1. The detection of the prey (sec), 2. The first attack (sec), 3. Successful predation (sec), and, in the case of successful hunting, the type of catch (catching the head, body or tail). Detection of the prey was recorded when the pikeperch turned in the direction of the prey, the first attack was recorded when the fish first began to swim toward the prey, and successful predation was recorded when the prey was captured (and finally consumed).

Based on the time points, we calculated the latency of detection (Ldet = TP detection-TP starting the experiment), the latency of the first attack (Lfa = TP first attack- TP starting the experiment), and the latency of predation (Lpred = TP hunting- TP starting the experiment).

### Statistical analyses

According to the Shapiro–Wilk test, our data were not normally distributed; therefore, nonparametric statistics were used. The distribution of successful hunting attempts, together with the comparisons of the hunting methods within groups and in the five tests was analyzed using chi-squared tests. A comparison of the exploratory behavior parameters of the two feeding groups was performed with the Mann–Whitney *U* test performed on the data of the 36 individuals. The correlation between the predation success and the latency of the first attack and the latency of the predation was calculated by Spearman correlation. The effects of exploratory parameters (Tmin, Dmin, and frequency), feeding aptitude (willingness to eat pellets), and the practice (five consecutive trials) were analyzed using a Cox Proportional Hazard regression model performed on the latency of the detection, first attack and predation measured in the total of 180 life foraging tests. Statistical analyses were performed using SPSS 17.0 software (SPSS 2008).

## Results

### Characterization of explorative behavior

The raw data of the novel object and the live prey foraging tests are presented in Table S1. We found that the frequency of exploring the new object was significantly higher among the pellet non-feeder group (Mann–Whitney *U* = 96, *P* = 0.034) even though neither the latency (Mann–Whitney *U* = 141.5, *P* = 0.515) nor the distance (Mann–Whitney *U* = 131.0, *P* = 0.323) of the closest approach was significantly different between the two groups (pellet non-feeder (NPF) and pellet feeder (PF)), yet the pellet non-feeder fish was slightly faster to explore the new object (Table 1).

**Table 1:** The distribution of time and frequency between the feeder (PF) and pellet non-feeder (PNF) groups during the novel object test

Parameter	Group (sample size)	Mean	SD	95% Confidence interval for mean	
				Lower bound	Upper bound
T minimum	PNF (*N* = 18)	12.7	7.7	8.9	16.6
	PF (*N* = 18)	15.0	9.0	10.5	19.5
	Total (*N* = 36)	13.9	8.3	11.0	16.7
D minimum	PNF (*N* = 18)	9.9	5.6	7.1	12.7
	PF (*N* = 18)	9.6	6.7	6.3	13.0
	Total (*N* = 36)	9.8	6.1	7.7	11.8
Frequency	PNF (*N* = 18)	4.7*	4.1	2.7	6.8
	PF (*N* = 18)	2.1*	2.5	0.8	3.4
	Total (*N* = 36)	3.4	3.6	2.2	4.7

*Means significant difference (*P* = 0.029), *PF* pellet feeder, *PNF* pellet non-feeder, *SD* standard deviation

### Predation success in the consecutive life-prey tests

The raw data of the experiment are presented in Table S1. 21 successful predators were detected, 11 of which belonged to the pellet non-feeder (PNF) group and 10 to the pellet feeder (PF) group. The 21 individuals consumed a sum of 47 prey items. No significant difference was found between the numbers of successful predations in PNF and PF (27 in PNF and 20 in PF out of 90 attempts; Chi^2^ = 1.411, df = 1, *P* = 0.234).

The predation success showed a correlation with the latency of the first attack (r = − 0.598, *p* < 0.001, *n* = 180) and the latency of the predation (r = − 0.985, *p* < 0.001, *n* = 180).

Of the successful individuals, 10 (6 in PF and 4 in PNF) preyed only once, 3 (2 in PF and 1 in PNF) twice, 4 (4 in PNF) three times, 1 (1 in PNF) four times, and 3 (2 in PF and 1 in PNF) individuals preyed five times.

### Factors influencing the latency of prey detection

**Fig. 1:**
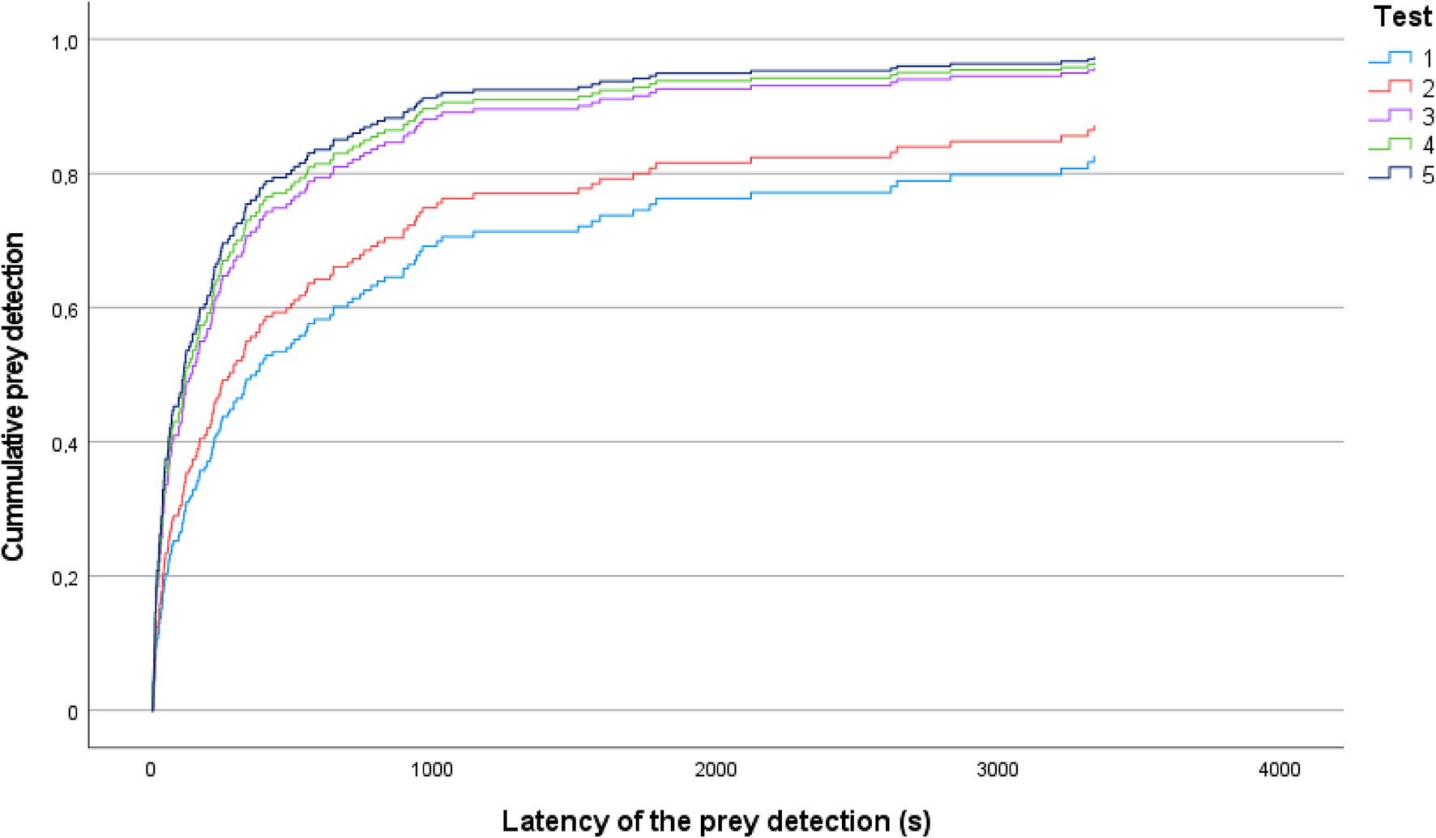
Cumulative event curves showing the effect of predation practice (five consecutive foraging trials) on the probability that fish had detected prey before a given time during an experimental trial

For the latency of prey detection, we found a significant effect of the two parameters of the novel object test measuring exploratory behavior, the minimum distance (Dmin) and the latency to achieving it (Tmin), but their hazard ratio moves around one, indicating only a small effect (Table 2). Besides, the predation practice (number of exposures to live prey) had also a significant effect on prey detection (Table 2). The mean latency of the prey detection was decreasing significantly in the first three consecutive tests (594.5 ± 924.9 s, 514.5 ± 784.4 s, and 349.4 ± 609.7 s in the first, second and third tests, respectively). However, the value of the fourth and fifth test (297.8 ± 345.9 s and 280.6 ± 467.1 s) was similar to the value measured in the third test (Fig. 1). The significance of the aptitude to pellet consumption was marginal (*P* = 0.055), resulting in a lower latency in the PNF group compared to the PF group (Exp(B) = 0.727, Table 2).

**Table 2:** Cox proportional hazards model testing for effects of exploratory behavior, aptitude to pellet consumption and predation practice on the latency of prey detection

Variable	*B*	SE	Wald	df	*P*	Exp (B)	95% CI for Exp (B)	
							Lower	Upper
**Dmin**	**0.032**	**0.013**	**6.035**	**1**	**0.014**	**1.032**	1.006	1.059
**Tmin**	**− 0.039**	**0.017**	**5.063**	**1**	**0.024**	**0.962**	0.929	0.995
Frequency	− 0.018	0.030	0.375	1	0.540	0.982	0.925	1.041
Feed	− 0.318	0.166	3.684	1	0.055	0.727	0.525	1.007
Test			**12.685**	**4**	**0.013**			
**Test (1)**	**− 0.726**	**0.255**	**8.113**	**1**	**0.004**	**0.484**	0.293	0.797
**Test (2)**	**− 0.564**	**0.251**	**5.050**	**1**	**0.025**	**0.569**	0.348	0.930
Test (3)	− 0.135	0.245	0.303	1	0.582	0.874	0.541	1.412
Test (4)	− 0.070	0.243	0.083	1	0.773	0.932	0.579	1.501

Significant effects are highlighted in bold. Overall model was significant (− 2 Log Likelihood = 1434.444, Chi square = 21.016, df = 8, *P* = 0.007)

*Dmin* minimum distance measured in the exploratory test, *Tmin* latency of achieving Dmin in the exploratory test, Frequency- number of approaches in the exploratory test, Feed- the aptitude to pellet consumption, Test- predation practice from the five consecutive trials, *B* regression coefficient, *SE* standard error, Wald- Wald chi-square, *df *degree of freedom, *P* significance, *Exp(B)* hazard ratio, *CI* confidence interval

### Factors influencing the latency of the first attack

In contrast to the latency of prey detection, the latency of the first attack was only affected by the frequency of the exploration with a marginal (*P* = 0.053) significance level, but not the minimum distance and its latency. In addition, the parameter was significantly affected by the aptitude to consume pellets (*P* = 0.005) and, similarly to the latency of detection, the foraging practice (*P* < 0.001) (Table 3).

**Table 3:** Cox proportional hazards model testing for effects of exploratory behavior, aptitude to pellet consumption and predation practice on the latency of first attack

Variable	*B*	SE	Wald	df	*P*	Exp (B)	95% CI for Exp (B)	
							Lower	Upper
Dmin	0.002	0.014	0.013	1	0.908	1.002	0.974	1.030
Tmin	− 0.027	0.021	1.642	1	0.200	0.974	0.935	1.014
**Frequency**	**− 0.067**	**0.035**	**3.733**	**1**	**0.053**	**0.935**	**0.874**	**1.001**
**Feed**	**− 0.559**	**0.201**	**7.728**	**1**	**0.005**	**0.572**	**0.386**	**0.848**
Test			**23.584**	**4**	**0.000**			
**Test (1)**	**− 1.388**	**0.315**	**19.452**	**1**	**0.000**	**0.250**	**0.135**	**0.463**
**Test (2)**	**− 0.991**	**0.288**	**11.861**	**1**	**0.001**	**0.371**	**0.211**	**0.652**
**Test (3)**	**− 0.525**	**0.268**	**3.831**	**1**	**0.050**	**0.592**	**0.350**	**1.001**
Test (4)	− 0.446	0.263	2.867	1	0.090	0.640	0.382	1.073

Significant effects are highlighted in bold. Overall model was significant (− 2 Log Likelihood = 1125.359, Chi square = 30.572, df = 8, *P* < 0.001)

*Dmin* minimum distance measured in the exploratory test, *Tmin* latency of achieving Dmin in the exploratory test, Frequency-number of approaches in the exploratory test, Feed- the aptitude to pellet consumption, Test- predation practice from the five consecutive trials, *B* regression coefficient, *SE* standard error, *Wald* Wald chi-square, *df* degree of freedom, *P* significance, *Exp(B)* hazard ratio, *CI* confidence interval

**Fig. 2:**
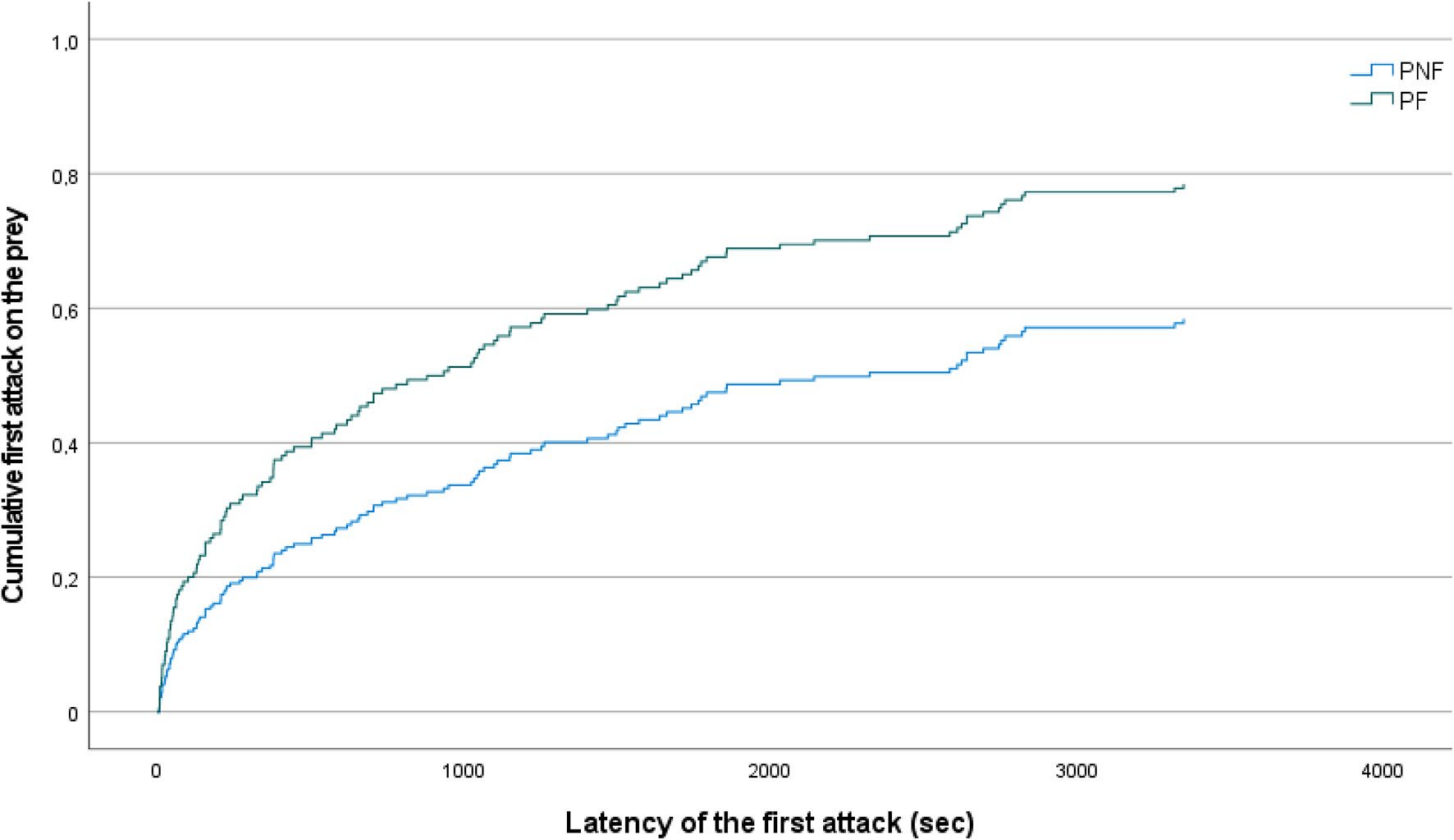
Cumulative event curves showing the effect of aptitude to pellet consumption (PF-pellet feeder, PNF-pellet non-feeder) on the probability that fish had first attacked the prey before a given time during an experimental trial

In the case of the PNF group, the latency was found to be lower compared to the PF group (Fig. 2; hazard ratio = 0.572, *P* = 0.005; average values: 1913.1 ± 1495.6 s in PF, *n* = 90 and 1546.1 ± 1470.5 s in PNF, *n* = 90).

**Fig. 3:**
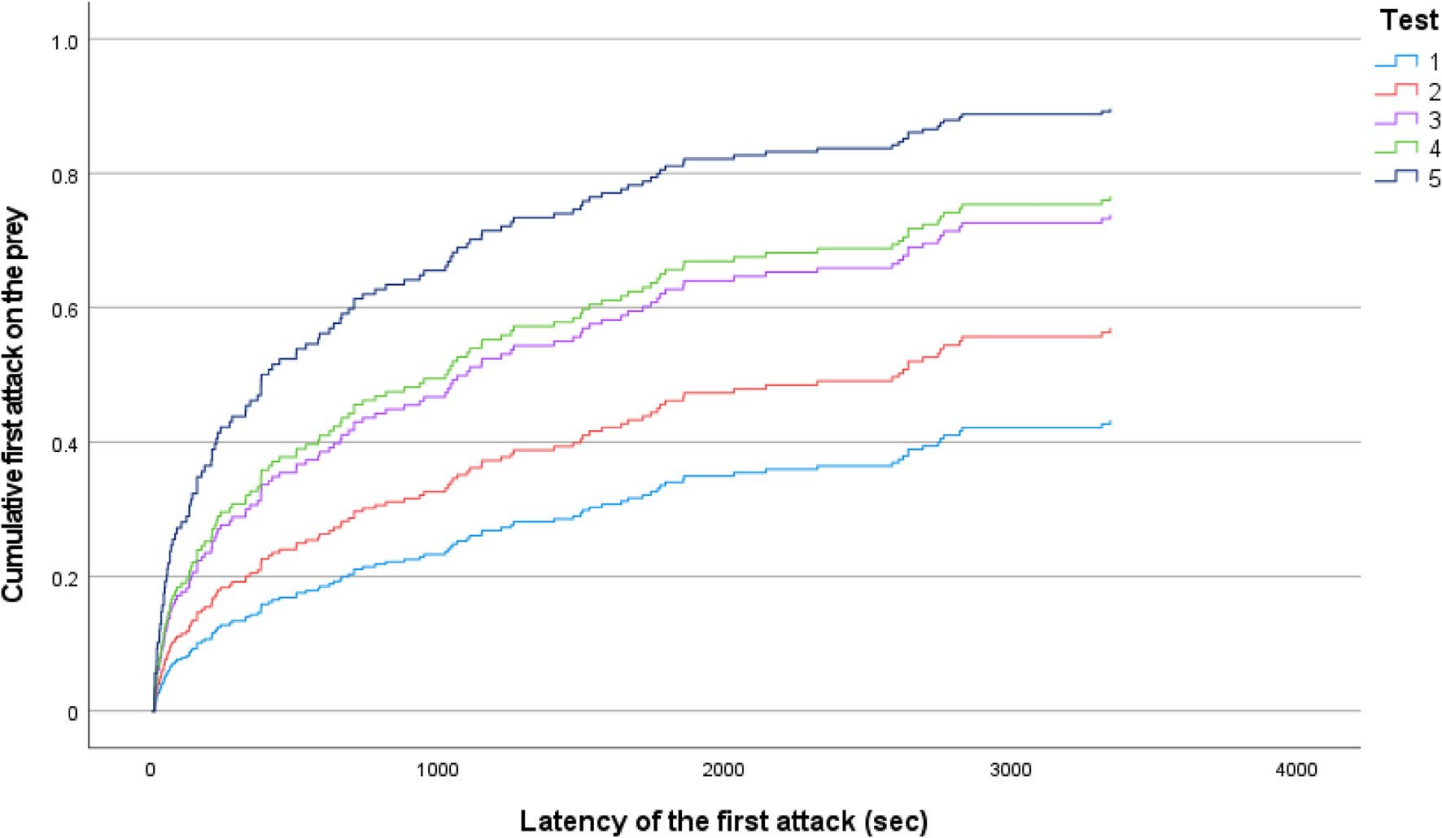
Cumulative event curves showing the effect of predation practice (five consecutive foraging trials) on the probability that fish had first attacked the prey before a given time during an experimental trial

The predation practice resulted in a significantly decreasing latency of the first attack. The change was significant in the first four consecutive trials (2408.1 ± 1485.0 s, 2096.6 ± 1497.5 s, 1625.9 ± 1500.4 s and 1500.5 ± 1322.7 s in the first, second, third and fourth trials, respectively). However, the latency value in the fifth trial was lower (1016.9 ± 1308.4 s) it did not differ significantly from the latency measured in the fourth trial (Table 3, Fig. 3).

### Factors influencing the latency of the successful predation

The latency of the successful predation was influenced by neither of the treatments in our experiment; the overall model was not significant (*P* = 0.385) (Table 4).

**Table 4:** Cox proportional hazards model testing for effects of exploratory behavior, aptitude to pellet consumption and predation practice on the latency of predation

Variable	*B*	SE	Wald	df	*P*	Exp(B)	95% CI for Exp (B)	
							Lower	Upper
Dmin	0.030	0.024	1.638	1	0.201	1.031	0.984	1.080
Tmin	− 0.012	0.034	0.128	1	0.721	0.988	0.925	1.055
Frequency	0.030	0.047	0.418	1	0.518	1.031	0.940	1.130
Feed	− 0.406	0.328	1.535	1	0.215	0.666	0.351	1.267
Test			4.979	4	0.289			
Test (1)	− 0.646	0.458	1.995	1	0.158	0.524	0.214	1.285
Test (2)	− 0.925	0.501	3.408	1	0.065	0.396	0.148	1.059
Test (3)	− 0.410	0.441	0.864	1	0.353	0.663	0.279	1.576
Test (4)	− 0.086	0.409	0.044	1	0.833	0.917	0.412	2.045

Significant effects are highlighted in bold. Overall model was not significant (− 2 Log Likelihood = 466.403, Chi square = 8.518, df = 8, *P* = 0.385)

*Dmin* minimum distance measured in the exploratory test, *Tmin* latency of achieving Dmin in the exploratory test, Frequency- number of approaches in the exploratory test, Feed- the aptitude to pellet consumption, Test- predation practice from the five consecutive trials, *B* regression coefficient, *SE* standard error, *Wald* Wald chi-square, *df *degree of freedom, *P* significance, Exp(B)- hazard ratio, *CI* confidence interval

### Type of predation in the experimental groups

The dominant type of successful predation was catching by the tail (40 × tail, 4 × body, and 3 × head). The distribution of the methods used in the five attacks did not differ (Pearson Chi^2^ = 5.804, *P* = 0.669; Table 1); however, the distribution of the catching methods marginally differed between the PF and NPF groups (Pearson Chi^2^ = 5.922, *P* = 0.052) caused by a complete absence of grabbing the body in the PNF group (Table 5).

**Table 5:** The distribution of foraging methods in the pellet feeder (PF) and pellet non-feeder (PNF) groups during the five consecutive trials

Group	Method	Test					
		1st	2nd	3rd	4th	5th	Total
PF	Tail	4	2	2	4	3	15
	Body	1	1	1	0	1	4
	Head	0	0	0	1	0	1
	Total	5	3	3	5	4	20
PNF	Tail	3	3	5	6	8	25
	Body	0	0	0	0	0	0
	Head	0	0	1	1	0	2
	Total	3	3	6	7	8	27

## Discussion

Although, in the live prey foraging test, the PNF group showed lower latency of prey detection and first attack, the two groups did not differ in the number and latency of successful predation. The exploration had an effect on the latencies of detection and first attack; the detection was influenced by the speed of the exploration, since the first attack by the frequency. Predation experience had a general effect on prey detection and latency of first attack. The complete absence of grabbing the prey body was observed in the PNF group resulting in a significant difference in the foraging methods compared to the PF group.

Our primary hypothesis was not confirmed as willingness for pellet consumption did not affect the predation success of the pikeperch in the tests which was 22% (20/90) in the PF and 30% (27/90) in the PNF group. In rainbow trout and brown trout, no differences were found in the predation success of hatchery stocks (18% and 16%); however, the prey consumption of wild fish was significantly higher in both species (82 and 81%) (Ward et al. 2018). Turesson et al. (2002) detected 26% predation success in pikeperch with a total length (TL) of 168–196 mm, which is similar to the value we measured. Another set of tests (Turesson and Brönmark 2004) experienced different predation successes in three predatory fish species. In perch *Perca fluviatilis* (TL 190–235 mm), 17.6% (35/199), and in pike *Esox lucius* (TL 182–221 mm), 15.1% (25/166); and in pikeperch (TL 192–228 mm), 8.5% (18/211) of the attacks ended with predation. Under natural conditions, the stomach content provides information on the previous 35–83 h of prey acquisition, depending on the temperature (Molnár and Tölg 1961). The stomachs of 77–91% of pikeperch with a body length of under 100 mm (plankton feeding stage) were full. This ratio dropped to 45–68% when changing to piscivory, and returned to the original value when the body length reached 500–800 mm (Specziár 2011).

In the successful predation trials of the present study, the average latency values of the different elements of the foraging behavior were 1.8, 5.6 and 10.6 min for the detection, first attack and predation, respectively. In the Turesson and Brönmark (2004) study on pikeperch, the latency until the first attack was around 4 min, and the predation took a further 13 min on average, showing similar values to the successful individuals in our study. Sundström and Johnsson (2001) found a longer latency of the first attack in hatchery individuals compared with wild ones in the brown trout, meaning that the wild fish were predating faster in the presence of other fish.

The dominant method of predation in our study was tail-first capture, and practice did not affect this tactic. In walleyes, *Sander vitreum*, both naive and experienced individuals used the tail-first method in 83% of cases attempt (Wahl et al. 1995). Turesson et al. (2002) investigated the hunting method and handling time in pikeperch of 168–196 mm in size and found that 78% (33/42) of the prey was grabbed tail-first, whereas prey handling time was shorter when head-first grabbing occurred. While studying cannibalism in pikeperch fingerlings, Colchen et al. (2019) revealed that in the early period until the age of 48 days post hatching, tail-first capture dominated; after that, the tactic changed to head-first. These authors hypothesized that this change could have been caused by the development of spiny rays in the conspecific prey larger than 20 mm. In this study, the traits of the prey were constant; accordingly, their alteration could not affect the method of capture. Further tests could use different (spiky) prey species to determine if negative experience influences the feeding tactic.

According to the novel object test, the frequency and speed of exploration were higher in the PNF group than in the PF group. Although both pellet consumption willingness and exploration influenced foraging behavior, their effects were different. Exploration (distance and latency) played a role in prey detection, with lower detection latencies in more exploratory individuals. However, pellet consumption willingness had only marginal effect on this parameter resulting in a lower latency in the PNF group. Fast exploring individuals should encounter new stimuli more quickly, may appear to be faster on learning challenges because of lower detection latency (Sih and Del Giudice 2012). In terms of latency of first attack, exploration had only a marginal effect, with more active individuals showing lower latency. However, the willingness to consume pellets had a strong effect on this parameter, with the PNF group having a latency of 80% of the PF group. These suggest that, although exploration and pellet consumption willingness have the same directional effect on the latency of the elements of foraging behavior, their effects seem to be independent of each other. In our previous investigation, the effect of exploration influenced, primarily, the time at which the consumption of alternative food (pellet) is attempted (Molnár et al. 2018). Regarding to the cognitive syndrome theory (Sih and Del Giudice 2012), explorative individuals have less accurate but faster decision-making. Sampling is favored when the cost of the delayed decision is lower, or the cost of making a wrong decision is high (Sih 1992). This can be observed during the transition, since the amount of natural food decreases, while the amount of alternative food increases during the process, and the risk of predation is low under the controlled conditions. Following the habituation process, the individuals avoiding pellet consumption were proactive and more explorative (Molnár et al. 2018). Pintor et al. (2014) found that in the case of pike the behavioral flexibility of the individuals in reversal learning could be measured as the switching time between the original and the new behavior. Their results suggested that flexible individuals are less efficient foragers (generalists) compared to specialists which could improve their skills on the few prey species with similar characteristics. As there are memory limits in the cognitive mechanism, the skills on the highly different prey types are not well transferable. In contrast to pellet consumption, foraging on live fish prey is an innate behavior. It seems that switching to this behavior favors specialists with fast decision-making and low accuracy. Ahlbeck and Holliland (2012) compared the exploratory behavior and the feeding on live prey, in the pikeperch reared in ponds and tanks. The pond-reared fish proved to be more active and started to feed on novel invertebrate prey (*Neomysis integer*) faster than the tank-reared conspecifics.

The hazard ratios pointed out the major role of the practice. For both the detection and the first attack latency, the individuals showed decreasing values in the first three or four consecutive trials. Many studies have shown that experience increases the efficacy of predation. In the Atlantic salmon *Salmo salar*, 6–15 consecutive attempts were needed to reach the maximal efficacy of predation as influenced by the natural conditions and social effects (Brown et al. 2003). In the brown trout, the latency of the first attempt also decreased during consecutive attempts (Sundström and Johnsson 2001). In the walleye, there was a significant difference in the time of predation during the first attempt between the naive and experienced fish, (as the attack time of experienced fish was only 5–10% of that of naive fish); however, the difference disappeared by the fourth attempt (Wahl et al. 1995). Based on our results, the learning curve of pikeperch is most similar to that of its northern American relative, walleye.

Although some elements of foraging behavior (detection and first attack latency) were influenced by exploratory behavior and aptitude to pellet feeding, none affected the latency of successful predation. Szopa-Comley et al. (2020) suggested that predation behavior reflects a ‘predator personality trait’ that is independent of other individual traits like body size, boldness and environmental factors and cannot be predicted from the study of axes of personality variation. Although our study could not detect a correlation between successful predation and exploration or willingness to consume pellets, repeatability was not defined for any of the behaviors, and it is more likely that other stronger effects mask the effects of the factors we examined. Successful predation depends largely on the behavior of not only the predator but also the prey (Belgrad and Griffen 2016). Individual predator activity and prey activity have an inverse relationship: active predator consume inactive prey and less inactive predator consume active prey individuals (Toscano et al. 2016; Andersson et al. 2021). Although in the present study, naïve individuals of one prey species were used, these individuals may show differences in their behavior.

Finally, it should be mentioned that our results were obtained by examining the behavior of solitary predators, which may result in a bias due to the lack of social interactions between the predator individuals. Naïve fish could modify their behavior through social learning. In pikeperch, this type of learning has controversial results in the habituation to pelleted food. Lepič et al. (2017) confirmed the positive effect of the teaching fish (*Vimba vimba*) on the adaptation of the pikeperch to the intensive culture. However, Horváth et al. (2013) did not find any effect in the case of the different predator species of teaching fish (*Perca fluviatilis*), while weaker habituation was observed in the case of the conspecific teaching individuals. In perch, the presence of conspecifics also significantly influenced the prey selection and, within this, the rate of cannibalism (thus predation hazard) as a function of the social behavior and activity of the predators (Andersson et al. 2021). Thus, it can be assumed that the presence of conspecifics can change the predation behavior also in the pikeperch, both through social learning and by changing the degree of predation risk (risk of cannibalism).

## Supplementary Information

Below is the link to the electronic supplementary material.

Supplementary file1 (XLSX 22 KB)

## Data Availability

All data generated or analyzed during this study are included in this published article and its supplementary information files.
